# Beyond individual willingness: family veto, legal knowledge, and the dynamics of organ donation in Türkiye

**DOI:** 10.55730/1300-0144.6365

**Published:** 2026-04-09

**Authors:** Hasan Giray ANKARA, Hakan DEĞERLİ, Semih BAŞ

**Affiliations:** 1 Department of Economics, Faculty of Economics and Administrative Sciences, Recep Tayyip Erdoğan University, Rize, Turkiye; 2 Department of Medical Services and Techniques, Vocational School of Health Services, Bilecik Şeyh Edebali University, Bilecik, Turkiye; 3 Department of Medical Services and Techniques, Vocational School of Osmaneli, Bilecik Şeyh Edebali University, Bilecik, Turkiye

**Keywords:** Organ donation decisions, family veto gap, family discussion, donation regulations, logistic regression model

## Abstract

**Background/aim:**

The aim was to understand the factors underlying the dynamics of organ donation behavior by focusing on three different aspects. Three models were tested in this regard, namely (i) individual willingness for the donation of one’s own organs, (ii) willingness to approve organ donation for close relatives, and (iii) a family veto gap.

**Materials and methods:**

A national-level adult sample from the Eurobarometer 72.3 survey (collected in 2009) conducted in Türkiye was utilized. Standard and penalized logistic regression models were employed to conduct the analyses.

**Results:**

The results show that discussing organ donation with family members and being aware of the organ donation law were associated with increased willingness to donate organs in all the study models. In addition, being a woman was associated with willingness to donate one’s own organs and approval for relatives, and living in an urban area was associated with approving donation for close family members. In the last model (the family veto gap) both family discussions and knowledge of the legal regulations were associated with a higher likelihood of discrepancy between personal willingness and family authorization decisions.

**Conclusion:**

Social communication and legal awareness have a pivotal role in shaping organ donation decisions in Türkiye. Therefore, health policies that encourage social conversations and raise public awareness regarding legal regulations on organ donation may help narrow the family veto gap and strengthen societal support for organ donation. Future studies could be beneficial by including cultural and religious factors and using longitudinal approaches to better capture how donation decisions evolve over time.

## 1. Introduction

Organ transplantation is extremely important because it offers life-saving and quality of life solutions for patients who are in the final stages of organ failure [1]. However, a significant problem is that the number of donated organs continues to be less than the need [2]. This problem threatens the lives of patients who need organ transplants [3] and also places a significant burden on the health systems of countries [4]. In order to address this problem, health policies that encourage organ donation are being implemented in various countries [5–7]. Many factors influence the success of such policies. This is because organ donation is not merely a medical process, but a complex social process encompassing individual, family, cultural, and legal dimensions [8]. These factors include individuals’ attitudes and behaviors regarding organ donation, age, sex, education level, religious beliefs, social norms, and family dynamics [9].

Although statistics show that organ transplants are increasing worldwide every year, the number of patients waiting for organs is much higher [10]. For example, according to the World Health Organization and the Global Observatory on Donation and Transplantation, a total of approximately 172,500 organ transplants were performed worldwide in 2023. This number represents a 9.5% increase compared to 2022.^1^ However, this number falls far short of the increase in patient waiting lists for organ transplants and the number of patients who die due to the lack of suitable organs [11]. For a statistic illustrating this widening gap, data from the Organ Transplant and Transplant Network shows that, in the United States, one person is added to the waiting list every 8 min and 13 people die each day while waiting because a suitable organ cannot be found.^2^ The situation in Türkiye is no different from the international one. As of May 2025, it is reported that 33,667 people were waiting for organ transplants and, although a total of 643,593 people had donor cards, only 5048 organ transplants had been performed.^3^ It is clear that, in Türkiye, organ donations fall far short of meeting the need for organ transplants [12].

In Türkiye, the defining legal framework regarding organ transplantation is regulated by Law No. 2238 on the Transplantation, Preservation, Transplantation, and Destruction of Organs and Tissues. According to this regulation, organ donation is subject to certain conditions. First, the individual must be 18 years of age or older, have sound mental health, and if the person has not made a declaration while alive, the consent of their relatives must be obtained. The law prohibits the trafficking of organs and tissues and details critical procedures such as determining medical death [13]. However, even if the deceased had an organ donation card, family members have the right to refuse organ donation under this law [14]. This situation negatively impacts organ donation rates in a society such as Türkiye, where family ties are strong, and is considered an important issue and area of research [15,16].

Organ donation is generally influenced by a variety of factors that affect an individual’s intentions and behavior towards donating [9,17,18]. In this context, the theory of planned behavior (TPB) and norm activation theory (NAT) are seen to be related to the subject in the literature [19,20]. The TPB argues that individuals’ behavior is influenced by subjective norms and perceived behavioral control, and that intentions are shaped accordingly [21]. In organ donation, an individual’s positive attitude towards donation, their relatives’ willingness to approve the donation, and their belief that the donation process is easy greatly influence their intention and behavior to donate [22,23]. On the other hand, NAT explains individuals’ positive social behaviors as the activation of personal norms [24]. This theory posits that when an individual recognizes that the consequences of a situation negatively affect others, they are more likely to exhibit positive social behavior [25]. Regarding organ donation, it is thought that if an individual is aware of the suffering experienced by patients on the organ waiting list and feels a personal responsibility in this matter, it can positively influence their decision to donate [26]. These theories are used to address behavioral influences on organ donation and to explain the key motivational factors that affect organ donation [9,15,23]. As aforementioned, the various factors influencing organ donation include social determinants such as age, sex, education level, socioeconomic status, and cultural background [27,28]. Cultural and religious beliefs are a critical factor shaping attitudes toward organ donation in Muslim-majority communities such as Türkiye [29,30]. Studies conducted in Türkiye have also shown that factors such as religious beliefs, cultural factors, and social awareness influence organ donation behavior [31,32].

Studies on organ donation have revealed the impact of donation-related family discussions and conflicts on donation behaviors and attitudes. These studies also address the moral and psychological tensions that arise in complex decision-making processes, such as veto situations within families [33–36]. During organ donation proceedings, a conflict can also arise between the deceased person’s wish to donate and the family’s opposition to this decision. This situation stems from differences in emotional and cultural sensitivities between families and individuals regarding bodily autonomy [37]. While it is suggested that family discussions about organ donation can reduce such conflicts, it can also create a family veto gap [38].

Previous studies using Eurobarometer 72.3 [39,40] have generally examined single intentions, such as donating one’s own organs or following legal considerations, at the macro level. However, there are very few studies in the literature that measure the asymmetry between an individual’s decision over their own body and their decision over a loved one’s body at the micro level. The present study fills this gap by reconstructing the existing data from a behavioral economics perspective. The aim was to provide a comprehensive insight into the growing literature on organ donation. Accordingly, it is sought to contribute by focusing not only on individual donation willingness, but rather on the approval of close relatives regarding organ donation and the family veto gaps. To ensure conceptual clarity, we define the family veto gap herein as the cognitive and behavioral discrepancy wherein an individual explicitly expresses a willingness to donate their own organs, yet simultaneously indicates a refusal or reluctance to authorize the organ donation of a deceased close relative.

## 2. Materials and methods

Three different approaches to organ donation were adopted: (i) individuals’ willingness to donate their own organs, (ii) their willingness to allow donations for close relatives, and (iii) family veto attitudes in order to offer a broader perspective. In doing this, the roles of family discussions and awareness of national legislation were specifically addressed. Drawing on established behavioral theories such as the TPB and NAM, and the moral psychology perspective on role conflict and decision-making under uncertainty, a comprehensive framework is provided for understanding how social, cultural, and organizational dynamics interact to shape donation outcomes.

In the present study, we employed the Eurobarometer 72.3 dataset. It is a cross-sectional-type survey conducted by the European Commission. The dataset includes various variables including sociodemographic characteristics and specially focuses on organ donation. Eurobarometer survey data are publicly available and anonymized; therefore, it is not necessary to obtain ethical approval to conduct the study [41]. While the Eurobarometer 72.3 data were collected in 2009, their selection for the present study was driven by their distinct structural alignment with our primary research objective. Specifically, this dataset offers a highly standardized and concurrent pairing of questions: it captures both an individual’s willingness to donate their own organs and their willingness to provide proxy consent for a deceased relative. This precise dual-question structure within a single national sample is essential for the empirical operationalization of the family veto gap. Furthermore, the cognitive dissonance and moral friction associated with surrogate decision-making (the veto gap) represent deep-seated structural human behaviors and cultural taboos regarding bodily integrity. Unlike general health literacy or daily campaign awareness, which fluctuate rapidly, this psychological discrepancy is a persistent structural phenomenon, making this historical dataset fundamentally relevant for modelling this specific behavioral gap.

The analyses involved three different models in the present study. Accordingly, the first model tries to explain the determinants of individuals’ intention to donate their own organs. In this model, the dependent variable is a dummy that takes a value of 1 if the participant is willing to donate their own organs; otherwise it is 0. The second model seeks the factors affecting the participant’s willingness to approve organ donation for close relatives. As in the previous model, the dependent variable employed in this model is a binary, taking a value of 1 if the person is willing to approve organ donation for a close relative; otherwise it is 0. The final model estimates the factors influencing the family veto gap. The interest variable is also coded as a dummy. This variable, which was not in the dataset, was created using the previous two dependent variables. Accordingly, observations where individuals are willing to donate their own organs but do not approve of a relative’s organ donation are coded as 1 in this variable. The opposite is coded as 0. In other words, the family veto gap is intentionally constructed post hoc. Cognitive dissonance regarding organ donation is a latent psychological friction. Directly asking respondents if they experience a veto gap would introduce severe priming and social desirability biases. By mathematically deriving this gap from two independent behavioral questions (willingness for self vs. proxy-consent for relatives), we capture the true, unprimed attitudinal discrepancy. Moreover, it is conceptually crucial to distinguish between a clinical family veto and the family veto gap operationalized in the present study. A true clinical veto is an interpersonal event occurring in a hospital setting where surviving family members override a deceased patient’s documented consent. In contrast, in the present study the intrapersonal, cognitive discrepancy is measured—where an individual supports personal donation but explicitly refuses to authorize it for a relative. While this survey-based discrepancy is not a direct measure of clinical veto rates, it serves as a critical behavioral antecedent and psychological proxy. If an individual exhibits this cognitive dissonance in a low-stakes survey environment, they represent the precise demographic profile that is highly likely to exercise a clinical veto in a high-stakes, emotionally charged hospital setting. Therefore, the family veto gap modelled here represents the underlying cognitive propensity that fuels the clinical phenomenon.

The key independent variables of interest were whether the participant had discussed organ donation with family members and whether the participant was aware of national organ donation laws. Both variables were coded in binary form. Accordingly, a value of 1 for the variables indicated that the participant had discussed organ donation with family members and was aware of national organ donation laws. A value of 0 indicated the opposite. Control variables include a wide range of variables. These include age (maintained in a continuous form), binary coded sex, marital status (i.e. married or single), place of residence (i.e. urban or rural), and occupational status (i.e. employed or unemployed). The selection of variables for the regression models was strategically guided by theoretical relevance, prior empirical literature, and methodological constraints. The key predictors—family discussion and awareness of the laws—were specifically selected to serve as proxies for the theoretical constructs of the TPB and NAT. Meanwhile, control variables such as age, sex, marital status, employment, and place of residence were included based on their consistently documented significance in previous organ donation studies. Finally, a strictly parsimonious approach was adopted for variable selection to prevent model overfitting and maintain an acceptable events-per-variable ratio, which is methodologically crucial given the rare-event structure of the dependent variable in model 3.

Observations with missing values in the variables included in the modelling were excluded, resulting in a final analysis sample of 752 individuals. The final analytical sample was reduced to 752 observations utilizing listwise deletion. While modern imputation techniques such as multiple imputation by chained equations (MICE) are often utilized in epidemiological modelling, they were deliberately excluded in the present study. Missing data in this dataset primarily stemmed from “don’t know” or “refusal” responses concerning the dependent variables (donation intentions). According to established econometric practices, imputing dependent variables introduces artificial noise and bias without adding genuine analytical information. Furthermore, nonresponses to deeply moral and ethical questions such as proxy organ donation are highly likely to be missing not at random, which directly violates the core missing at random assumption required for MICE. Therefore, listwise deletion was the most methodologically rigorous approach to restrict the analysis solely to respondents who had formed definitive cognitive stances. A preliminary attrition check indicated no severe systematic deviations in the primary sociodemographic baseline (e.g., sex distribution) between the retained and excluded samples. Descriptive statistics of the variables used in the models are presented in Table 1.

Three different regression models were established in the study to examine the determinants of organ donation decisions and the difference in family veto. The first model is a multivariate logistic regression aimed at revealing the effect relationship between an individual’s own intention to donate organs. The second model also uses multivariate logistic regression to predict organ donation consent for close relatives, employing the same set of predictors. The first and second model estimates provide insight into relational dynamics and potential moral conflicts by allowing the identification of differences in donation decisions between oneself and another person—a relative. The third model deals with the family veto gap. Given the small number of respondents exhibiting this behavior, Firth’s penalized logistic regression was used to reduce small-sample bias and address potential separation issues, ensuring more reliable coefficient estimates. All analyses were also performed using the survey weighting provided in the Eurobarometer dataset to ensure representation of the Turkish population.

To ensure the validity of the estimates, several model diagnostics were performed. Multicollinearity was assessed using the variance inflation factor (VIF). The mean VIF was well below the threshold of 5 (mean VIF = 2.14), which confirms that multicollinearity was not an issue. Model discrimination was evaluated using the area under the receiver operating characteristic (ROC) curve (AUC). The AUC values were 0.64 for model 1 and 0.69 for model 2, indicating acceptable predictive power. For model 3, Firth’s penalized logistic regression was employed due to the rare-event structure of the data. It is seen that this approach served as a robustness check to address potential small-sample and separation biases.

## 3. Results

The results table (Table 2) presents the estimated associations between the explanatory variables and the three outcomes of interest. The first column lists all explanatory variables included in the models. Model 1 reports the estimated results for individuals’ willingness to donate their own organs, model 2 reports the estimated results for respondents’ authorization of organ donation for close relatives, and model 3 reports the results for the family veto gap. In Table 2, both logistic regression coefficients (log-odds) and marginal effects are presented. The logistic coefficients are reported to show the direction and statistical significance of the variables. However, the interpretation of log-odds is not straightforward for policy implications. Therefore, marginal effects were calculated and reported alongside them. It is seen that marginal effects provide clear effect sizes, showing the percentage-point changes in the probability of each organ donation behavior. In model 1, sex, family discussion about organ donation, and legal awareness were positively associated with a person’s willingness to donate their own organs. Accordingly, being female was found to be associated with an 11% increased probability of wanting to donate one’s own organs. However, having discussed organ donation with family members was associated with approximately an 18% higher probability of making the decision to donate organs, while awareness of national organ donation legislation was associated with an increase of approximately 27%. In other words, female participants were more likely to donate organs and participants who had spoken to their families about organ donation or were aware of the national legislation were also more likely to donate.

In the second model, which focused on consent for organ donation by close relatives, being female was also found to increase the probability of consent, by 9%. Additionally, living in an urban area was associated with an 11% increase. Similarly, family discussions increased the probability of approval by 15%, while awareness of the regulations increased the likelihood of consent for donation by relatives by 30%. Overall, these findings demonstrate that both sociodemographic characteristics and factors such as information or social involvement are associated with donation decisions for others. Similar factors were also found to be influential in this model, with the exception of the location of residence.

In the last model, examining the family veto gap, discussing organ donation with family members was associated with a 44% increased likelihood of showing a discrepancy between the individual’s own will and the consent of relatives. Awareness of the national legal regulations regarding organ donation was associated with a 45% increased likelihood of a family veto gap.

Figure illustrates the estimated marginal effects of all explanatory variables across the three models. Points represent the predicted change in the probability of each outcome associated with each variable and error bars indicate the corresponding confidence intervals. It provides a clear visual comparison of the relative strength and direction of associations for individual donation willingness, authorization for relatives, and the family veto gap. The chart is included to visually summarize the associations between explanatory variables and the three organ donation outcomes. By plotting the estimated marginal effects with confidence intervals, the figure allows readers to quickly assess both the magnitude and direction of each variable’s association across models. This visual presentation makes it easier to compare the relative importance of factors influencing individual willingness, approval for relatives, and the family veto gap, highlighting patterns that may be less immediately apparent in tabular results. Overall, the chart enhances interpretability and provides a concise, intuitive overview of the key findings.

## 4. Discussion

The present study makes a significant contribution to the literature by examining organ donation behaviors in the context of Türkiye through a three-dimensional approach, including individual donation intention, family consent for donation, and the family veto gap. The results of the study highlight the role of family discussions and legal awareness in attitudes towards organ donation, using a national dataset from a developing country. In addition to the individual’s willingness to donate, which has been the focus of previous research [9,17,18], the present study offers a comprehensive perspective by considering both the broader perspective (i.e. relative consent and veto gap) and the family dynamics and legal framework issues in organ donation. Previous studies using this dataset have mainly examined whether individuals are willing to donate their own organs. However, focusing only on personal willingness ignores an important real-world barrier: family refusal. By constructing the family veto gap variable, in the present study a specific human conflict is measured—when an individual is willing to donate their own organs but would not approve the same decision for a close relative. Capturing this gap reveals that a person’s willingness to be a donor does not automatically mean they will give consent for a family member. This contribution to the literature suggests that policymakers should not only encourage individuals to become donors, but also address the emotional difficulty of making that decision on behalf of a relative.

The study results show that family discussions and legal awareness are positively associated with both individual attitudes towards organ donation and consent for donation among relatives. Therefore, this supports the idea that the decision to donate organs is not merely an individual choice, but also a social process in which family communication plays a critical role. Therefore, it can be interpreted within the framework of the TPB, which suggests that individual behaviors are guided by intentions and shaped by attitudes, subjective norms, and perceived behavioral control [21]. In line with this result, family discussions about organ donation serve as a powerful mechanism for reinforcing subjective norms; individuals perceive that family members support donation, which in turn reinforces their own intention to donate. Individuals are more likely to donate organs when they feel that their immediate environment accepts and supports such an act [22,23]. Indeed, many studies in the literature highlight results consistent with this [42–45]. For instance, a study on the behavior of potential organ donor families in the UK after the implementation of the Organ Donation (Default Consent) Act noted the impact of family interviews on their decision-making processes [42]. A study comparing donor and nondonor families in Türkiye highlighted the impact of intrafamily discussions on decision-making processes [14]. Another study suggests that the difficulty of talking about death in Türkiye prevents families from discussing organ donation, which in turn lowers donation rates [43].

The present study shows that awareness of the legal framework regarding organ donation can positively influence individuals’ trust in the donation process. This result underscores the importance of public awareness, particularly on sensitive issues where legal regulations can appear complex, such as organ donation [44]. In the context of the TPB, it can be argued that being informed about legal regulations can improve perceived behavioral control by reducing uncertainty surrounding the organ donation process and increasing individuals’ confidence that the process is transparent and manageable. This is consistent with previous research highlighting that the perceived ease of donation procedures significantly influences the intention to donate organs [22,23]. From the perspective of NAT, the impact of family discussions and organ donation-related legal awareness can be interpreted as dynamics that activate a person’s moral norms, because NAT emphasizes that social behaviors such as organ donation arise from individuals becoming aware of the consequences of inactivity on others and taking personal responsibility for those consequences [24,25]. Discussing organ donation within the family helps individuals confront the moral and emotional dimensions of this act, increasing their awareness of the societal need for organs and the ethical responsibility of contributing.

The results also show that women are more likely than men to intend to donate their own organs and to give their consent to the organ donation of their relatives. Puoti et al. [45] state that women are more likely to donate organs than men due to their greater sense of responsibility and altruism. Yee et al. [46] also found that women are more likely to donate their own organs compared to men. Along with this, in the same study, it was determined that women are motivated by both the wishes of the family member and the belief that the deceased no longer needs the organs when it comes to organ donation of deceased family members, whereas men tend to prioritize the wishes of the family member more [46]. In studies conducted in Türkiye, it has been found that women are significantly more likely to donate their own organs compared to men [47,48]. However, when the consent rates of family members in cases of cadaveric organ donation were examined, it was found that the consent rates for male cadavers were higher than those for female cadavers [31].

The findings of the present study indicate that individuals living in urban areas are more likely to approve organ donation for their close relatives compared to those living in rural areas. Studies examining the effects of settlement location on organ donation generally focus on individuals’ attitudes toward their own organs, while research on the donation of organs by close relatives is limited. In this context, it has been concluded that individuals living in urban areas are more inclined to donate their own organs compared to those in rural areas [48–51], and the likelihood of approving organ donation for close relatives is at a similar level of association [52,53].

One result of the present study is that family discussion and legal awareness increase the likelihood of a family veto gap. This suggests that family discussions and legal awareness confront individuals with an internal conflict between individual autonomy (the deceased’s will) and family authority (legal veto rights) [15,16], and that this situation also highlights the decision-making gap. Indeed, Liu et al. [38] found that an individual’s willingness to donate their own organs is higher compared to the willingness of family members to donate their organs, supporting this finding of our study. According to Sque et al. [54], family members experience a profound struggle when balancing the desire to maintain the physical integrity of their deceased loved one against the selflessness of providing the “gift of life”; in this context, the sacrifice is perceived as a distressing loss of bodily wholeness. Consequently, it is explained that even when individuals are willing to donate their own organs, they refrain from granting permission for the organ donation of their family members. This situation can also be evaluated within the framework of the TPB and NAT. The TPB posits that behaviors are shaped by individual attitudes, subjective norms—such as social and familial expectations—and perceived behavioral control [21]. According to the results, while an individual’s general attitude toward organ donation may be positive, the norm of preserving a family member’s bodily integrity can suppress the intention to donate. On the other hand, NAT posits that moral norms are activated when individuals recognize that their inaction may cause harm to others and subsequently assume personal responsibility [25]. Consistent with the findings, the desire of individuals to donate their own organs can be attributed to the activation of prosocial norms focused on saving lives. Yet, in the context of family organ donation, the moral obligation to protect the bodily integrity of a loved one often takes precedence over the altruistic sacrifice of saving an unknown person. Consequently, this dynamic can impede the activation of altruistic norms as conceptualized by NAT, as highlighted by Sque et al. [54].

The finding that family discussions and legal awareness increase the likelihood of a family veto gap may seem counterintuitive at first. However, this result points to an asymmetric behavioral response. While communication and legal knowledge accelerate an individual’s willingness to donate their own organs, they do not overcome the emotional and moral burden of proxy-consent at the same rate. It is frequently noted that surrogate decision-makers tend to be more risk-averse and emotionally defensive when making choices concerning a loved one’s body, compared to their own. Therefore, the gap widens not because awareness reduces surrogate consent, but because personal consent rises much faster, leaving a cognitive discrepancy. It is understood that generalized awareness campaigns are not sufficient to break down the psychological barriers associated with proxy decision-making. However, there are also studies in the literature that have found that communication established within the family regarding organ donation positively influences both the individual’s decisions to donate their own organs and their willingness to give consent for the donation of their close relatives’ organs [55]. Leblebici [56] stated that in Türkiye the refusal rates of families to donate organs are high and that family consent is of great importance. Similarly, Yeniocak and Ergin Özcan [57] stated that even if a patient in Türkiye had an organ donation card, their organs could not be used without the consent of their relatives. Within the TPB framework, this gap can be seen as a conflict between individual attitudes and the influence of subjective norms, where family attitudes may restrict how an individual expresses their intentions to donate. From a NAT perspective, it may represent a moral inconsistency: while individuals’ personal norms support giving, conflicting family values or emotional distress weaken the activation of these norms at the moment of actual decision-making.

In conclusion, the present study offers a different perspective in the literature by providing comprehensive evidence on organ donation behaviors in Türkiye, a developing country. The multifaceted nature of donation decisions is examined by analyzing individual donation willingness, relative consent, and family veto deficits within three models. The results highlight the importance of awareness of family discussions and national legislation; both were associated with more generous attitudes across the models.

The study’s strengths include integrating the multifaceted aspects of organ donation into a single framework and the use of predominantly nationally representative survey data. Methodological robustness is demonstrated by the application of Firth’s penalized logistic regression. However, the study has several limitations that should be critically acknowledged. First, the cross-sectional design of the data strictly limits our ability to establish causal inference between the predictors and organ donation behaviors. Second, relying on self-reported measurements for sensitive moral topics makes the data susceptible to social desirability bias, where respondents may overstate their altruistic intentions. Third, the study may be subject to potential selection bias, as individuals who choose to participate in such comprehensive surveys might systematically differ in their civic engagement or health awareness compared to nonparticipants. Fourth, the inherent limitations of secondary data analysis, including the older vintage of the Eurobarometer 72.3 dataset (collected in 2012), restrict the scope of the study. Although the cognitive dissonance between self-donation and proxy-consent represents deeply rooted structural behavior rather than a fast-moving trend, relying on an older dataset limits our ability to capture very recent shifts in public opinion or contemporary legal changes. For instance, the Eurobarometer dataset does not include specific measures for health literacy or prior clinical exposure to organ donation. Finally, this constraint, combined with the rare occurrence of the family veto gap in model 3, required a parsimonious model design to maintain an acceptable events-per-variable ratio and avoid overfitting. Consequently, there is a potential bias introduced by omitted variables, as theoretically important factors such as specific religious affiliations, educational attainment, or detailed income brackets could not be directly included, although they may be partially captured through proxies such as awareness of the laws and urban residence. Future studies using primary data should aim to include these sociocultural and health literacy variables to build more comprehensive models.

For decision-makers in health policy and management, the study findings indicate that interventions to increase organ donation should go beyond individual-level awareness campaigns and actively promote family communication and legal literacy. To operationalize these findings, specific and actionable strategies are required. First, regarding target populations, communication strategies must specifically target the designated next-of-kin and surrogate decision-makers who bear the ultimate moral responsibility, rather than solely focusing on the individual potential donor. Second, concerning intervention strategies, public health campaigns should pivot from the standard message of register as a donor to a more relational directive: discuss your donation decision with your family. Additionally, clinical protocols should be developed to provide specialized grief counselling for families at the critical moment of surrogate decision-making to alleviate the emotional burden that drives the veto gap. Finally, regarding implementation pathways, these targeted family discussions can be structurally integrated into the primary healthcare system (e.g., via family physicians during routine check-ups) and national digital health infrastructures (e.g., Türkiye’s e-Nabız system) to systematically notify and educate the designated next-of-kin.

Through the development of these specific policies, the likelihood of families, who play a critical role in organ donation, exercising their veto power can be reduced and societal support for organ donation can be strengthened. For researchers, future studies should leverage these insights by testing designs that incorporate longitudinal data and model diverse cultural and religious factors in real-world contexts.

## Figures and Tables

**Figure:**
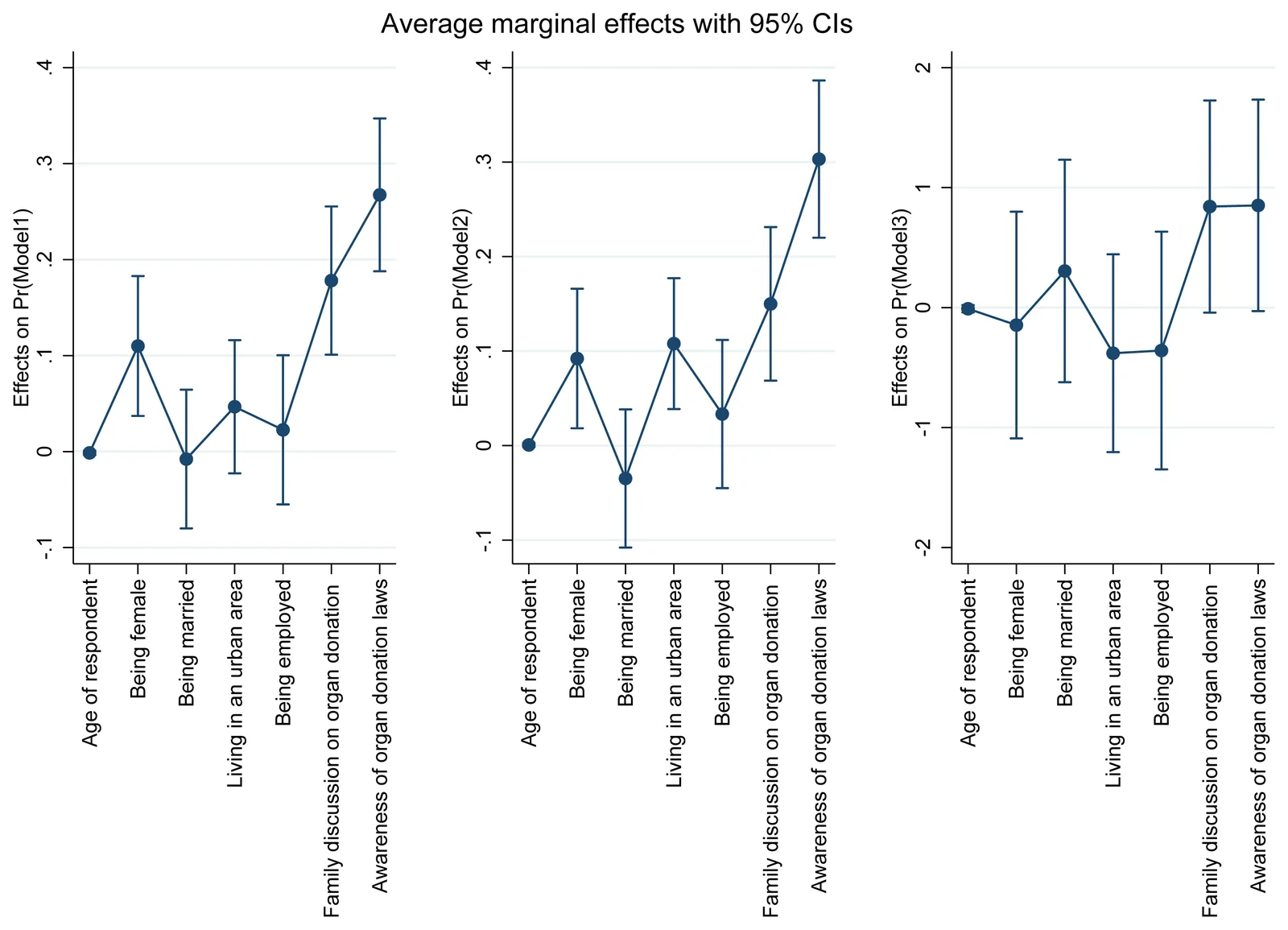
Estimated marginal effects with respect to explanatory variables across organ donation models.

**Table 1:** Descriptive statistics.

Variables descriptions	Obs.	Mean	Min	Max
**Dependent variables**				
Organ donation intention	752	0.415	0	1
Approval of organ donation for close relatives	752	0.452	0	1
Family veto gap	752	0.032	0	1
**Control variables**				
Age	752	36.273	15	85
Female	752	0.533	0	1
Male*	752	0.467	0	1
Married	752	0.62	0	1
Single*	752	0.38	0	1
Urban residence	752	0.64	0	1
Rural residence*	752	0.338	0	1
Employed	752	0.342	0	1
Unemployed*	752	0.658	0	1
**Key independent variables**				
Family discussion	752	0.229	0	1
Legal awareness	752	0.194	0	1

* Reference category

**Table 2:** Factors associated with organ donation behaviors.

Variables	Logistic regressions		Penalized logistic regression	Marginal effects		
	Model 1	Model 2	Model 3	Model 1	Model 2	Model 3
Age	−0.006 (−0.017, 0.004)	0.003 (−0.008, 0.014)	−0.01 (−0.040, 0.021)	−0.001 (−0.004, 0.0009)	0.001 (−0.002, 0.003)	−0.009 (−0.040, 0.021)
Female	0.521*** (0.169, 0.872)	0.426*** (0.08, 0.772)	−0.146 (−1.09, 0.799)	0.11*** (0.037, 0.183)	0.092** (0.018, 0.166)	−0.078 (−1.090, 0.798)
Married	−0.037 (−0.379, 0.305)	−0.161 (−0.500, 0.178)	0.305 (−0.622, 1.231)	−0.008 (−0.080, 0.064)	−0.349 (−0.108, 0.038)	0.154 (−0.622, 1.231)
Urban residence	0.221 (−0.109, 0.551)	0.499*** (0.172, 0.826)	−0.38 (−1.204, 0.444)	0.047 (−0.023, 0.116)	0.107*** (0.039, 0.177)	−0.171 (−1.224, 0.453)
Employed	0.107 (−0.261, 0.475)	0.154 (−0.209, 0.517)	−0.358 (−1.348, 0.632)	0.023 (−0.055, 0.100)	0.033 (−0.045, 0.112)	−0.173 (−1.441, 0.722)
Family discussion	0.843*** (0.459, 1.226)	0.694*** (0.306, 1.081)	0.842* (−0.042, 1.726)	0.178*** (0.101, 0.255)	0.149*** (0.069, 0.231)	0.437*** (−0.012, 1.526)
Legal awareness	1.266*** (0.851, 1.681)	1.403*** (0.973, 1.832)	0.852 (−0.029, 1.732)	0.267*** (0.188, 0.347)	0.303*** (0.220, 0.386)	0.452*** (−0.038, 1.822)
Constant	−0.995*** (−0.102, −1.236)	−0.991*** (−1.777, −1.221)	−0.924*** (−1.868, −1.737)			
Observations	*752*	*752*	*752*	*752*	*752*	*752*

* p<0.10,

** p<0.05,

*** p<0.01

## Data Availability

The data that support the findings of this study are available from the data repository: https://search.gesis.org/research_data/ZA4977, https://doi.org/10.4232/1.11140

## Footnotes

1 Global Observatory on Donation and Transplantation (2024). International Report on Organ Donation and Transplantation Activities 2023 [online]. Website https://www.transplant-observatory.org/ [accessed 29 Sep 2025]

2 Health Resources & Services Administration. Organ Donation Statistics (2025). Organ Donation Statistics [online]. Website https://www.organdonor.gov/learn/organ-donation-statistics [accessed 29 Sep 2025].

3 Turkish Transplant Foundation. Statistics (2025). Organ Bağışı İstatistikleri [online] Website https://www.tonv.org.tr/tr/organ-bagisi/istatistikler/ [accessed 29 Sep 2025].
